# Abscess of Right Corpus Striatum—Irritation and Inflammation of the Membranes of the Brain, with Effusion of Lymph at the Base—Absence of Paralysis, or Other Symptoms, until within Twelve Hours of Death

**Published:** 1849-09

**Authors:** 


MANCHESTER PATHOLOGICAL SOCIETY.

*Abscess of right Corpus Striatum—irritation and inflammation of the membranes of the brain, with effusion of lymph at the base—Absence of paralysis, or other symptoms, until within twelve hours of death.*—Mr. Farr presented the brain of a young woman, 19 years old, of which the right corpus striatum and parts adjacent were broken down and excavated by chronic abscess. Extensive effusion of lymph and serum had taken place into the ventricles. On section through the corpus striatum, upwards of an ounce of grass-green colored pus, mixed with softened brain, flowed out. The convolutions were flattened, as from too great compression. The arachnoid covering the hemispheres was dry. The pia mater had not the appearance of congestion. The dura mater adhered with unusual firmness to the skull. Concrete lymph was effused into the sub-arachnoid cellular tissue, around the medulla oblongata, pons varolii, cerebellum, and optic nerves. The girl had syphilis, but with this exception all the other viscera of the body were healthy.

She was a prostitute, and had been admitted into one of the sick wards of the Manchester union workhouse, suffering from diarrhœa. She was addicted to drinking, had a small appetite, and bore a reputation for cheerfulness and liveliness of disposition.

On the night preceding her death, she retired as usual, but became restless, and frequently got in and out of bed without assigning any reason for the act. Sickness and diarrhœa set in, and at nine o'clock in the morning convulsions of the entire body occurred, and lasted a quarter of an hour. The right eyelid remained closed, and the pupils were dilated and disobedient to light. At ten o'clock the convulsions returned, the right arm on this occasion being more agitated than the left; breathing slow and slightly blowing: pulse quick and feeble; skin of natural warmth. By six o'clock she was perfectly insensible, and at seven she died.

Attention was called to the previous history of the girl, and the known fact of her cheerfulness up to the day of admission, and subsequently.

The diarrhœa under which she suffered differed in nothing from the severe autumnal purging then so common all over Manchester. The first clue to the disease in the brain showed itself in the restlessness and getting in and out of bed,—a symptom noticed by Mr. John Bell in connection with abscess of the brain following injuries of the skull. The convulsions, dilated pupils, and the apoplectic coma that followed, seemed to receive an explanation on the acknowledged principle that they may be, and indeed frequently are, caused by pressure, or the irritation that follows the tearing of the white fibres of the brain; and it is presumable that on the morning of these occurrences the abscess of the corpus striatum gave way into the ventricles.

The dryness of the membranes covering the cerebral hemispheres, the lymph effused around the base, and the flatness of the convolutions, bespoke alike the pressure and irritation that had been silently endured.

Perhaps the most remarkable feature of the case was the abscess in the corpus striatum, which failed to produce any recognised symptoms by which the least suspicion of disease could be aroused, although it must have existed some time.

As it is customary now to look upon the corpora striata as the anterior cerebral ganglia of the spinal chord, through which the white fibres of the brain pass from the hemispheres, and descend to form the motory tract, receiving a stimulus in their passage through these bodies, it would seem conformable to physiology that so serious a pathological lesion should have been more manifest. To quote from Mr. Solly—"I believe it is an invariable fact, that extravasation into the corpus striatum is followed by paralysis, and consequently there is no portion of the brain that pathology has so clearly indicated the function of, as the corpus striatum, in so far as volition and the production of voluntary motion is concerned." Other pathologists have rendered like testimony.—*London Medical Gazette.*

